# Persons with Chronic Spinal Cord Injury Have Decreased Natural Killer Cell and Increased Toll-Like Receptor/Inflammatory Gene Expression

**DOI:** 10.1089/neu.2017.5519

**Published:** 2018-08-01

**Authors:** Paige Herman, Adam Stein, Katie Gibbs, Ilya Korsunsky, Peter Gregersen, Ona Bloom

**Affiliations:** ^1^ The Feinstein Institute for Medical Research, Northwell Health.; ^2^ Department of Physical Medicine and Rehabilitation, Zucker School of Medicine at Hofstra Northwell.; ^3^ Robert S. Boas Center for Genomics & Human Genetics, The Feinstein Institute for Medical Research.; ^4^ Department of Molecular Medicine, Donald and Barbara Zucker School of Medicine at Hofstra Northwell, Northwell Health, Hempstead, NewYork.

**Keywords:** genomics, human studies, inflammation, spinal cord injury

## Abstract

Infections are the leading cause of death for individuals with traumatic spinal cord injury (SCI). Along with increased infection rates, inflammation is often also observed in persons with chronic SCI. Together, immunological changes post-SCI are also poised to impede neurological recovery and mediate common medical consequences of SCI, including atherogenesis and neuropathic pain. The molecular mechanisms contributing to increased infection susceptibility and inflammation in persons living with SCI are poorly understood. Here, we used tools of functional genomics to perform a pilot study to compare whole-blood gene expression in individuals with chronic SCI (≥1 year from initial injury; *N* = 31) and uninjured individuals (*N* = 26). We identified 1815 differentially expressed genes in all SCI participants and 2226 differentially expressed genes in persons with SCI rostral to thoracic level 5, compared to uninjured participants. This included marked downregulation of natural killer cell genes and upregulation of the proinflammatory Toll-like receptor signaling pathway. These data provide novel mechanistic insights into the causes underlying the symptoms of immune dysfunction in individuals living with SCI.

## Introduction

There are an estimated 17,000 new traumatic spinal cord injuries (SCIs) each year in the United States and approximately 353,000 Americans living with SCI.^1^ Despite advances in acute and chronic care, the life expectancy for individuals with SCI is significantly below that of able-bodied individuals.^1^ Infections are the leading cause of death for persons with SCI, who are more than 80 times more likely to die of sepsis than able-bodied individuals.^2,3^ Infections (genitourinary or respiratory) are also the leading cause of rehospitalization post-SCI.^4,5^ The molecular basis for infection susceptibility in persons with SCI is incompletely understood. Increasing evidence also supports a link between increased infection susceptibility and poor outcome in persons with SCI. Infections (pneumonia and post-operative wound) were identified as the first independent risk factor for poor neurological outcome post-SCI, particularly in persons with injuries rostral to thoracic level 5 (T5), the spinal level where sympathetic nervous system (SNS) fibers exit the spinal cord and innervate organs of the immune system.^6–14^ This is particularly important in light of the fact that most injuries occur rostral to the level of T5.^1^

Another immunological alteration post-SCI is systemic inflammation, which has been observed in both pre-clinical and clinical studies.^13,15^ Kwon and others have demonstrated elevated systemic inflammatory mediators in persons with acute SCI, where some appear to correlate with injury severity and/or neurological recovery.^16–21^ We and others have identified elevated systemic inflammatory proteins in persons with chronic SCI.^19,22–26^ Given that inflammatory mediators promote many common medical conditions in able-bodied persons, (including insulin resistance, accelerated atherogenesis, neuropathic pain, and metabolic syndrome), it is reasonable to postulate that inflammation plays a role in these comorbid conditions in persons with SCI as well.^27–31^

Other immune system alterations have been observed in persons with chronic SCI, particularly in natural killer (NK) cells, which are innate immune cells that play a critical role in killing infected cells and tumor cells, as well as secreting cytokines that modulate other immune cell subsets. NK cells are particularly responsive to aerobic exercise, which triggers their mobilization from the spleen into the circulation.^32,33^ In 1994, Nash showed that in comparison to able-bodied persons (*N* = 8), blood-derived CD3-CD56^+^ NK cells were reduced in number and in cytotoxicity in persons with chronic quadriplegia and that both could be improved by 30 min of aerobic exercise (*N* = 8).^34^ Campagnolo and colleagues showed that the total white blood cell count was comparable in persons with chronic quadriplegia and able-bodied persons (*N* = 5, 5), but the percentage of lymphocytes (but not monocytes or granulocytes) was elevated and the percentage of NK cells was reduced.^35^ In a subsequent larger study of persons with chronic SCI at levels rostral to (*N* = 26), caudal to (*N* = 10) T5, and able-bodied persons (*N* = 34), there were no differences in numbers of total white blood cells, monocytes, lymphocytes, granulocytes, basophils, or eosinophils, but there was a decrease in the percentage of NK cells and an increase in CD4^+^ T cells.^36^ A few other studies with small group sizes have also provided evidence of reduced NK cytotoxicity and reduced NK cell numbers in persons with SCI, although the relationship of these findings to injury level or severity has been inconsistent, likely attributed to heterogeneous and small sample sizes.^34–36^

Despite the direct clinical relevance of understanding the molecular basis for alterations in the immune system of persons with chronic SCI, we are aware of only one previous study investigating gene expression in immune cells of persons with chronic SCI. In that study of men (*N* = 13) with chronic SCI, elevated levels of BCMA, BAFF, and APRIL, which are autoimmunity-promoting cytokines, were identified in circulating leukocytes.^37^ Here, in an effort to broaden our understanding of the mechanisms underlying altered immune system function in persons with chronic SCI, we performed a pilot study to determine and compare whole-blood transcriptional profiles of individuals living with chronic SCI (*N* = 31) and uninjured (*N* = 26) individuals.

## Methods

### Participants

This prospective, observational pilot study was performed in an academic medical center in accord with the ethical standards of and approved by the Northwell Health IRB (protocol #09-026B). Inclusion criteria were: ≥18 years old; history of SCI at any level; traumatic SCI ≥1 year previously; and injury classification of American Spinal Injury Association Scale (AIS) grade of A-D, as determined by a physiatrist board certified in SCI medicine. Exclusion criteria were: concurrent infection such as frank urinary tract infection as indicated by lab evidence (urinalysis, positive culture) and some clinical occurrence such as hematuria, fever; incontinence between catheterizations; and pressure ulcers, cancer, chemotherapy, neutropenia, or autoimmune disease. Uninjured participants were ≥18 years old, without history of SCI, within an age range and sex distribution similar to the chronic SCI participants. Clinical and demographic data are found in Table 1. Participants with SCI were requested to have two blood draws performed 6 months apart and a majority (*N* = 24 of 31) completed two study visits.

**Table 1.** Summary of Clinical and Demographic Characteristics

*All participants (*n*)*	*Uninjured*	*Chronic SCI*	______
	26	31	
Age (mean ± SEM, range)	48 ± 2 (23–66)	55 ± 3 (21–80)	*p* < 0.05
Sex (male)	19 (73%)	25 (81%)	
Chronic SCI participants: Data are presented as *n* (%)^a^ or mean (SEM)			
Mechanism of injury			
Fall	10 (32)		
MVA	7 (23)		
Sports	10 (32)		
Other	4 (13)		
AIS grade			
A	16 (52)		
B	2 (6)		
C	4 (13)		
D	9 (29)		
Level			
Cervical	18 (58)		
Thoracic	11 (36)		
Lumbar	2 (6)		
≥T5 and above	23 (74)		
≤T6 and below	8 (26)		
Years from injury			
(mean ± SEM, range)	15.7 ± 2, (1–44)		

^a^ Percents may not add up to 100%, attributed to rounding.

SEM, standard error of the mean; SCI, spinal cord injury; MVA, motor vehicle accident; AIS, American Spinal Injury Association Impairment Scale.

### Microarray gene expression profiling

RNA was isolated from whole-blood in a Paxgene tube, using standard methods and the manufacturer's protocol (Qiagen QIAcube, Venlo, The Netherlands). RNA quality and quantity was measured using the Bioanalyzer (Agilent Technologies, Santa Clara, CA). RNA was amplified using the Illumina RNA Total Prep Amplification Kit (Life Technologies, Carlsbad, CA) and analyzed on the HT-12v4 Expression BeadChips (Illumina, San Diego, CA), which contained 47,323 probe sets. Raw data were background subtracted and quantile normalized in Genome Studio (Illumina), and missing bead values were imputed, with the k-Nearest Neighbor algorithm used by Genome Studio. There were 34,694 unique gene symbols, and expression data were averaged over multiple probe sets for the same gene symbol. Data exported from Genome Studio was log_2_ transformed for analysis in Partek Genomics Suite (Partek Inc., St. Louis, MO). In Genomics Suite, genes that failed to have a transformed maximum value ≥5 in any sample were filtered from the dataset, resulting in 11,261 genes analyzed. Principal component analysis (PCA) was performed in Partek Genomics Suite to examine overall variation in gene expression profiles using default settings in two dimensions (first two components). In the SCI group, we used repeated-measures analysis of variance (ANOVA; Partek Genomics Suite) to determine that overall, visit 1 and visit 2 gene expression profiles did not differ significantly from each other (see Results). PCA identified visit 1 as an outlier for 2 participants (Partek Genomics Suite), so visit 1 data were used for 29 participants and visit 2 data were used for 2 participants. Differential expression of transcripts was determined by one-way ANOVAs between SCI and AB groups, using the step-up method of Benjamini and Hochberg to correct *p* values with a false discovery rate (FDR) = 0.05 (Partek Genomics Suite).^38^ To determine expression profiles according to immune cell types, modular analysis was performed as done by Chaussabel and colleagues.^39,40^ Computational deconvolution was performed using the Hematology Expression Atlas (“*HaemAtlas*”) pipeline in CellMix, using Benjamini and Hochberg corrected *p* values, with an FDR = 0.05.^41,42^ Analysis of the functions of differentially expressed genes was performed using an open bioinformatics platform, *Enrichr*.^43^

## Results

### Study cohort

Participants in this pilot study were persons with chronic SCI (*N* = 31) or were uninjured (*N* = 26), were mostly male and were of similar ages (*p* < 0.06; Table 1). Participants with SCI were injured for 15.7 ± 2.3 years (mean ± standard error of the mean [SEM]). The etiologies of injury were: fall (32%), motor vehicle accident (MVA; 23%), sports (32%), and other (13%). Among study participants, the most common AIS grades were A and D (*N* = 16, 9, respectively). SCIs were neurologically complete (AIS A) in 52% of the participants and occurred mostly rostral to thoracic level T5 (74%; Table 1).

### Gene expression analysis

**FIG. 1.**
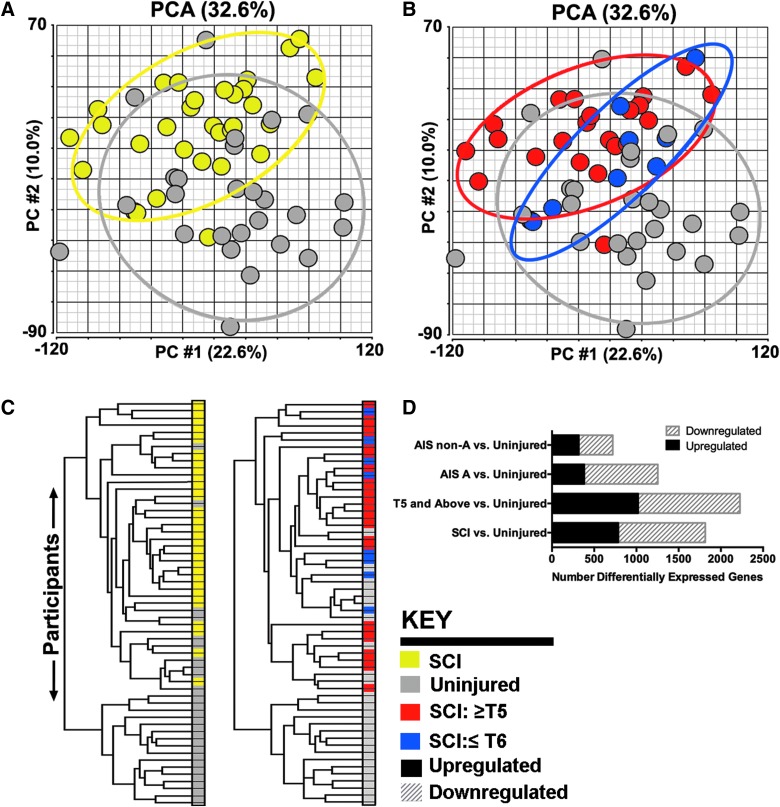
Chronic spinal cord injury influences whole-blood gene expression. (**A** and **B**) Principal component analysis (PCA) illustrates that the first two components explain 32% of variations in gene expression among participants (*n* = 11,261 filtered genes). Ellipses on PCA plots are drawn with a standard deviation of 2. Circles represent individual participants. Colors in the key indicate the same participant designated by SCI status (A) or injury level (B). (**C**) Dendrograms from two-way hierarchical clustering of participants by differentially expressed genes: (left) clustering of all participants (*n* = 1815 genes); (right) clustering of participants SCI at levels rostral to T5 versus uninjured participants (*n* = 2226 genes). (**D**) The number of differentially expressed genes (FDR of 0.05, Benjamini and Hochberg adjusted) between groups is shown. Key for colors in panels are indicated. AIS, American Spinal Injury Association Impairment Scale; FDR, false discovery rate; SCI, spinal cord injury.

Our first question was how variable overall gene expression was in persons living with chronic SCI. For most participants (*N* = 24 of 31) with SCI, data were obtained from two blood samples obtained 6 months apart and potential differences in gene expression were analyzed using a repeated-measures ANOVA (RM-ANOVA; Partek Genomics Suite). Visits 1 and 2 in participants with chronic SCI were not significantly different from each other (RM-ANOVA; FDR = 0.05). Therefore, a single sample from each participant with SCI was used for all subsequent analyses presented, as described in Methods. Major variations in gene expression by participant group were determined using PCA (Fig. 1). The first two components explained 32.6% of the total variation in gene expression (Fig. 1A,B). PC2 (10% of variance) is most associated with injury status, SCI versus uninjured.

Although this pilot study was not designed to examine differences in gene expression according to individual variables in SCI, we explored changes in gene expression by injury level, dichotomized as rostral or caudal to T5, where SNS disruption occurs. When the injury level of participants with SCI was indicated on the PCA plots, it revealed that most participants with higher cord-level injuries were distributed around a mean centroid that was more distant from that of uninjured controls, compared with that of SCI participants with lower levels of injury (caudal to T5; Fig. 1B). No notable difference in participant distribution was observed by sex, although females were the minority in each group (as is the case for SCI in general), or by injury severity, as indicated by AIS grade (Supplementary Table 1) (see online supplementary material at http://www.liebertpub.com).

Hierarchical clustering of differentially expressed genes demonstrates overall differences in participants with SCI versus uninjured participants (Fig. 1C). Dendrograms show clustering that differentiates participants with SCI from most uninjured participants, with a few exceptions (Fig. 1C). When gene expression profiles of participants were compared by injury level (Fig. 1C), this bifurcation was essentially preserved. We identified 1816 genes that were significantly differentially expressed between all SCI and uninjured participants (FDR = 0.05), of which 785 were upregulated and 1031 were downregulated in the group with SCI (Fig. 1D; Supplementary Table 1) (see online supplementary material at http://www.liebertpub.com). As mentioned earlier, 74% of participants in this study were injured rostral to T5 and 52% were AIS A, so we did not have a sufficient number of participants to observe significant differences in gene expression between subgroups of SCI participants by injury level or severity, after correcting for multiple hypotheses. We therefore compared gene expression of SCI with uninjured participants. Participants with SCI at higher levels (rostral to T5) had the greatest number of differentially expressed genes compared to uninjured participants, of which 1021 were upregulated and 1205 were downregulated (Benjamini and Hochberg adjusted FDR = 0.05; Fig. 1D; Supplementary Table 1) (see online supplementary material at http://www.liebertpub.com). Participants with neurologically complete injuries (AIS A) had 1257 differentially expressed genes compared to uninjured participants (Benjamini and Hochberg adjusted FDR = 0.05; Fig. 1D; Supplementary Table 1) (see online supplementary material at http://www.liebertpub.com). Participants with less neurologically complete injuries (AIS B–D) had 720 differentially expressed genes compared to uninjured participants (Benjamini and Hochberg adjusted FDR = 0.05; Fig. 1D; Supplementary Table 1) (see online supplementary material at http://www.liebertpub.com). Taken together, these results demonstrate that chronic SCI broadly influences the expression of genes in circulating leukocytes.

### Characterization of differentially expressed genes

**FIG. 2.**
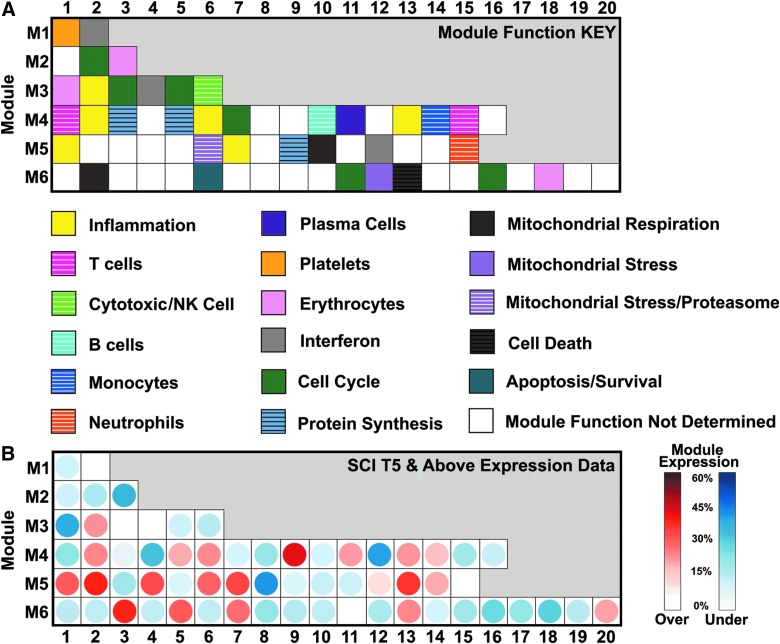
Modular analysis of genes differentially expressed in participants with spinal cord injuries at levels rostral to T5. The modular analysis was performed as described by Chaussabel and colleagues. (**A**) Grid and corresponding key below indicate the name of each module of genes. (**B**) Grid shows the percentage of genes within each module that are over- or underexpressed in participants with SCI at levels rostral to T5 compared to uninjured participants. The color of the circle within each module indicates the directionality of expression change for the majority of genes within the modules (red = overexpression, blue = underexpression). The color intensity scale corresponding to the percentage of over- or underexpression is shown to the right. NK, natural killer; SCI, spinal cord injury.

To better characterize the immunological phenotype of participants with SCI at levels rostral to T5, we next utilized a modular approach developed by Chaussabel and colleagues to analyze coordinately expressed clusters of genes in transcriptional profiles in whole-blood (Fig. 2).^39,40^ Module identity is based on empirically clustered gene expression from eight different autoimmune and infectious diseases, and module level indicates the conservation of coexpression across the number of diseases (eight in total) used to build the modules.^39,40^ Modules were named according to the known roles of transcripts within them, whereas others remain “undetermined,” because their functional relationship is not yet known (Fig. 2A). Participants with SCI at levels rostral to T5 showed underexpression of modules related to many cell types, including NK cells (M3. 6), B cells (M4.10), T cells (M4.1, M4.515), and platelets (M1.1; Fig. 2B). Erythrocyte modules (M2.3, M3.1, and M6.18) were downregulated, although there was no clinical indication of anemia in the medical charts of participants (data not shown). Other downregulated modules included cell cycle (M2.2, M3.5, and M6.16, but not M9.42, which was upregulated) and mitochondrial respiration (M6.2, M5.10). Strikingly, all of the six distinct inflammation-related modules (M3.2, M4.2, M4.6, M4.13, M5.1, and M5.7), a mitochondrial stress/proteasome module (M5.6), and a monocyte module (M4.14) were overexpressed in participants with SCI at levels rostral to T5 (Fig. 2B). The plasma cell module was upregulated (M4.11), and the cell death module (M6.13) was also upregulated. Individual genes changed within each module and the percentage of genes changed within a module is shown in Supplementary Table 1 (see online supplementary material at http://www.liebertpub.com). Additional modules whose function has yet to be designated were also significantly changed (e.g., M4.9, M5.13, M6.3, M6.5, and M6.7). These modules included genes such as the chemokine receptor CCR2 (M4.9), regulators of the nuclear factor kappa B (NF-κB) pathway (M5.13), and IBTK, a tyrosine kinase inhibitor expressed in adaptive immune cells (M6.7), among others (Supplementary Table 1) (see online supplementary material at http://www.liebertpub.com). Because functional characterization of the modules is ongoing, we expect that more information will be yielded from this analysis in the future.

Of course, changes in transcriptional profiles of a mixed cell population as done here may simply reflect changes in the relative abundance of the immune cell types. In past modular analysis studies, gene expression data reflected relative abundance of cell types by flow cytometry and complete blood count (CBC).^44,45^ Therefore, we estimated relative proportions of immune cell types in participants with SCI at levels rostral to T5 versus uninjured participants examined in the modular analysis, using methods of computational deconvolution.^46,47^ Briefly, publicly available gene expression data from reference populations of immune cell subsets (monocytes, B and T lymphocytes, and NK cells) are used to deconvolve data obtained from a mixture of immune cell subsets.^47^ Here, we used the Hematology Expression Atlas (“HaemAtlas”) pipeline in CellMix to estimate relative proportions of immune cell types in participants with SCI at levels rostral to T5 versus uninjured participants.^41,42^ By this method, we did not find significantly different estimated frequencies of populations of CD14^+^ monocytes, CD4^+^ and CD8^+^ T cells, CD56^+^ NK cells, erythroblasts, CD19^+^ B cells, megakaryocytes, and CD66^+^ granulocytes between SCI at levels rostral to T5 and uninjured participants (Benjamini Hochberg adjusted *p* < 0.05; Supplementary Table 1) (see online supplementary material at http://www.liebertpub.com).

### Natural killer cell gene expression is reduced in participants with high-level chronic spinal cord injury

**FIG. 3.**
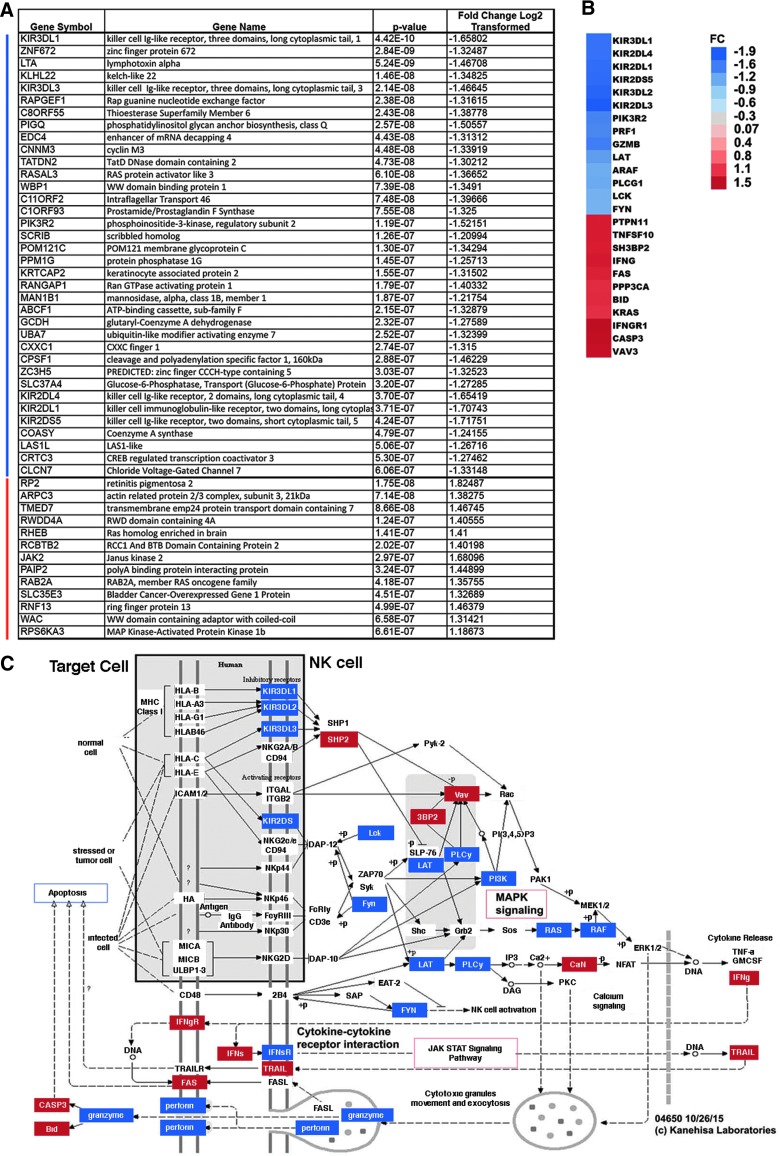
Functional enrichment analyses show reduced NK cell genes in participants with high-level SCI. (**A**) Table shows the top 50 differentially expressed genes by significance for participants with SCI at levels rostral to T5, as compared to uninjured participants. Gene symbol, gene name, *p* values, and fold change (log2 transformed) are shown in indicated columns. Blue and red lines (left) indicate genes downregulated and upregulated in participants with SCI at levels rostral to T5, as compared to uninjured participants. (**B**) Heatmap displays average fold change values for all differentially expressed genes in the KEGG category “Natural Killer cell mediated cytotoxicity.” Expression key for fold change (FC) for the log2-transformed expression data is shown, with expression values log transformed. (**C**) The KEGG pathway map (map04650) “NK cell pathway” is adapted here from http://www.kegg.jp/kegg/kegg1.html, with differentially expressed genes in participants with SCI at levels rostral to T5 shown in red (upregulated), blue (downregulated), and gray (not significantly different). The KEGG database has been described previously.^58–60^ KEGG, Kyoto Encyclopedia of Genes and Genomes; NK, natural killer; SCI, spinal cord injury.

Among the top 50 significantly differentially expressed genes in participants with SCI at levels rostral to T5, 36 were downregulated (Fig. 3A). As noted above, although NK cells numbers have previously been reported to be decreased in chronic SCI, the deconvolution approach did not allow us obtain evidence of lower levels of CD56^+^ NK cells. Nevertheless, it is notable that 5 of the 36 most significantly downregulated genes were killer inhibitory receptors most commonly found on NK cells (Fig. 3A,B). Reduction in NK cell gene expression was also detected in the cytotoxic/NK cell module described above (M3.6; Fig. 2B). The Kyoto Encyclopedia of Genes and Genomes (KEGG) bioinformatics platform independently identified the “Natural Killer cell mediated cytotoxicity” pathway as significantly enriched in genes differentially expressed in participants with SCI at levels rostral to T5 (Fig. 3C).

### Toll-like receptor signaling and inflammatory gene expression is increased in participants with high spinal cord-level spinal cord injury

Among the top 50 upregulated significantly differentially expressed genes in participants with SCI at levels rostral to T5 were a member of the protein tyrosine Janus kinase family (JAK2), a member of the Ras oncogene family (RHEB), and a mitogen-activated protein kinase (MAPK)-activated protein kinase (RPS6KA3/MAPK activated protein kinase 1b; Fig. 3A). Other genes related to proinflammatory JAK-STAT signaling that were differentially expressed in individuals with SCI compared to uninjured individuals included: AHCTF1, BCL2, CCND3, CISH, IFNGR1, IL2RB, IL10RB, IL11RA, IL15, NEIL2, PIAS4, PIK3R2, PTPN11, PTPN22, SOCS4, TIPRL, and TYK2 (Supplementary Table 1) (see online supplementary material at http://www.liebertpub.com).

Several upregulated differentially expressed genes in participants with SCI at levels rostral to T5 have previously been shown to be elevated at the protein level in persons with acute or chronic SCI. In individuals with acute SCI, Kwon and colleagues found that S100A9 levels in cerebrospinal fluid correlated with injury severity.^17^ Here, we observed elevated S100A9 in participants with chronic SCI by injury level or severity (Supplementary Table 1) (see online supplementary material at http://www.liebertpub.com). In more recent proteomics studies by Kwon and colleagues, ENO2, PRDX2, ALDOC, and B2M were among 27 factors identified as candidate circulating metabolic biomarkers of injury severity in persons with acute SCI.^21^ Intriguingly, these factors were also significantly differentially expressed, with the same directionality, in the present data set from persons with chronic SCI, either by injury level or severity (Supplementary Table 1) (see online supplementary material at http://www.liebertpub.com). We also confirmed the past study of gene expression profiling in persons with chronic SCI, as the autoimmunity-promoting factor TNFRSF17/BCMA is also elevated in this data set (Supplementary Table 1) (see online supplementary material at http://www.liebertpub.com).^37^

**FIG. 4.**
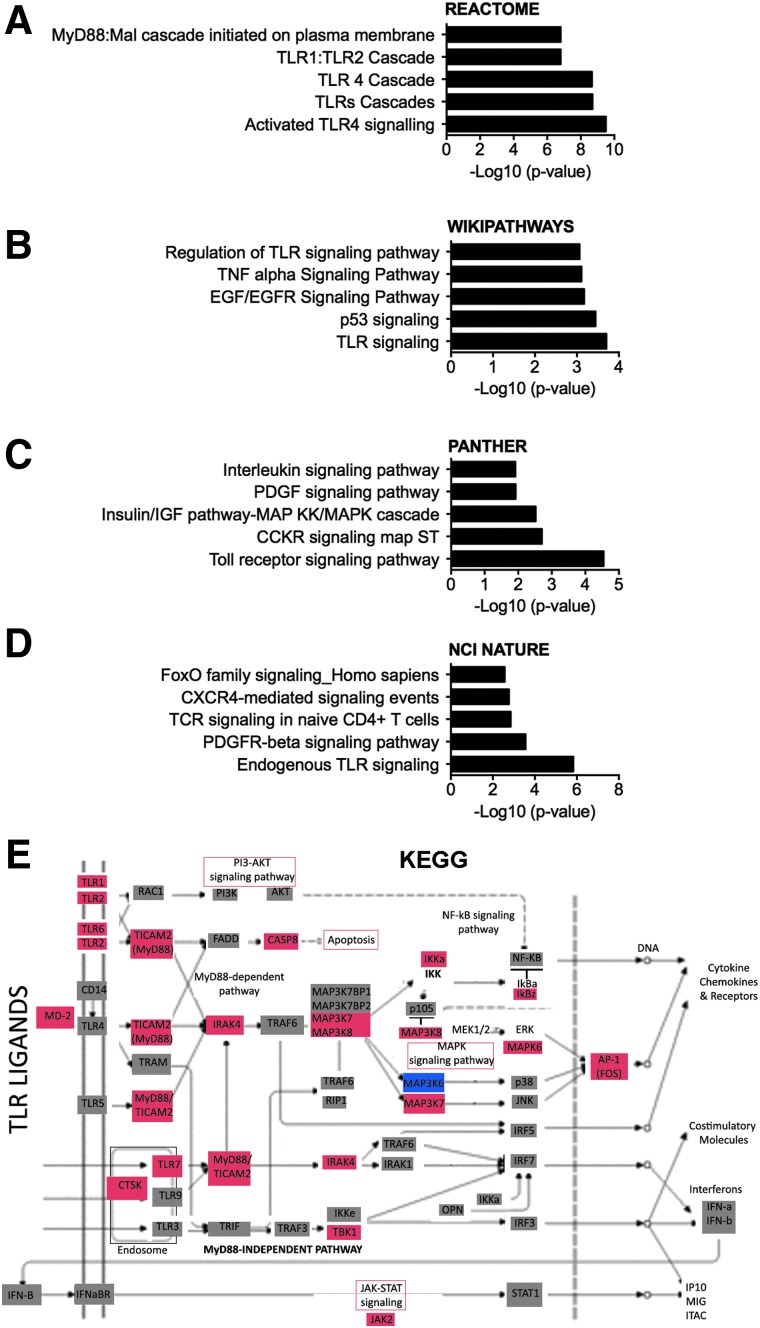
Pathway analyses of upregulated differentially expressed genes in participants with spinal cord injuries at levels rostral to T5. Bar graphs indicate significantly enriched categories of upregulated genes in participants with SCI at levels rostral to T5 identified by Reactome (**A**), WikiPathways (**B**), Panther (**C**), and NCI Nature (**D**) databases. Categories shown are significant with *p* ≤ 0.05. (**E**) The KEGG pathway map (map04620) “Toll-like receptor signaling” is adapted here from http://www.kegg.jp/kegg/kegg1.html, with differentially expressed genes in participants with SCI at levels rostral to T5 shown in red (upregulated), blue (downregulated), and gray (not significantly different). KEGG, Kyoto Encyclopedia of Genes and Genomes; SCI, spinal cord injury; TLR, Toll-like receptor.

We next used several bioinformatics pathway analysis platforms to characterize the function of upregulated differentially expressed genes in participants with SCI at levels rostral to T5. Strikingly, multiple independent platforms, including Reactome, WikiPathways, PANTHER, NCI Nature, and KEGG, identified Toll-like receptor (TLR) signaling pathways as the most highly enriched pathway (Fig. 4A–E). We also detected elevated levels of the endogenous TLR ligand, high-mobility group box 1 (HMGB1; Supplementary Table 1) (see online supplementary material at http://www.liebertpub.com).

## Discussion

Most of the participants in this study were male, had chronic SCIs that were neurologically complete (AIS A), and were at levels rostral to T5. These characteristics reflect the national data for people living with SCI.^2^ We found clear evidence that whole-blood gene expression is broadly changed in persons living with SCI, which we presume to be the result of changes in expression by more than one cell type (Figs. 1 and 2). Compared to uninjured persons, the greatest number of differentially expressed genes was in persons with SCI at levels rostral to T5, where SNS outflow from the spinal cord to immune and other organs would most likely be altered, which was also the largest subgroup of participants with SCI in this study (Fig. 1D). As mentioned earlier, we did not observe dramatically different gene expression profiles by AIS grade, but this is likely because of the smaller number of participants in AIS B (*N* = 2), C (*N* = 4), and D (*N* = 9) subgroups (see Fig. 1D; Supplementary Table 1) (see online supplementary material at http://www.liebertpub.com). In the comparison of uninjured participants and participants with SCI at levels rostral to T5, most of the differentially expressed genes were downregulated, with enrichment among NK-cell–related genes. These data support the importance of revisiting the functionality of NK cells in individuals with chronic SCI and their potential relevance to increased infection susceptibility, given that three previous studies have shown decreased percentages of NK cells, with reduced NK cell cytotoxicity in two of three studies.^34–36^ Using computational methods, we did not estimate reduced number of NK cells in persons with SCI rostral to T5 as observed by Nash and Campagnolo, but CBC or flow cytometry are more direct measurements of this and we will incorporate these methods in future studies. Alternatively, differences between the past human NK cell studies and the data presented here may derive from heterogeneity among the participants within the relatively small numbers of subjects. Although there have since been several studies with mixed results exploring changes in NK cells after exercise in persons with SCI, there are few mechanistic studies of NK cells in animal models of SCI.^33^ In athymic nude rats, which do not have T lymphocytes, NK cell markers were elevated intraspinally at 8 weeks post-injury, whereas motor recovery was only improved at 1 week post-injury, compared to Sprague-Dawley rats.^48^

Inflammation related modules were highly upregulated. Although participants with clinically significant infections were excluded from this study (see Methods), the directionality of gene modules that were significantly changed (Fig. 2) reflects data similar to able-bodied individuals with infections, likely reflecting an increased inflammatory response.^44,45,49,50^ We also noted elevations in mitochondrial stress, which has been linked to inflammation in other populations, such as patients with early-stage heart disease, where decreased mitochondrial respiration correlated with TNF-alpha, interleukin (IL)-6, and CRP levels in peripheral blood mononuclear cells (PBMCs).^51^

The enrichment in TLR signaling revealed by pathway analyses in the upregulated differentially expressed genes in persons with SCI at levels rostral to T5 is also consistent with elevated inflammation. TLRs are pattern recognition receptors expressed by immune cells, where their activation triggers activation of transcription factors like NF-kB and AP-1, which, in turn, promotes production of inflammatory cytokines, chemokines, and costimulatory molecules.^52,53^ Whereas most widely studied in monocytes and other antigen presenting cells, different immune cell types express different combinations of TLRs, both constitutively and after stimulation with TLR ligands.^52,53^ Human blood NK (CD3^–^ CD56^+^) cells express multiple TLRs (TLR1–9), but currently there is disagreement over the expression (protein and mRNA) and subcellular localization (surface or intracellular) of individual TLRs within NK cell subsets in whole-blood or cultured *ex vivo* in naïve or stimulated conditions.^54,55^ Several studies have demonstrated that in response to exposure to TLR ligands, NK cells cultured with IL-15/-18 produce proinflammatory cytokines, such as interferon (IFN)-gamma.^54,55^ NK cells from blood of sepsis patients that were cultured with IL-15/-18 had reduced production of IFN-gamma compared to NK cells isolated from healthy controls.^55^ Because here we present data of elevated IFN-gamma and IFN-gamma receptor expression in whole-blood (Fig. 3B), future studies of gene expression in purified individual cell types are needed to address the cellular source(s) of IFN-gamma in persons with chronic SCI.

Studies of systemic inflammation in animal models of general trauma showed that TLR decreased responses of spleen cells depended on TLR4 and TLR9, whereas TLR2 contributed to decreased spleen cell proliferation.^56^ Intraspinal TLR4 levels are elevated in a rat model of spinal cord ischemia, and TLR4 signaling promotes blood–brain barrier leakage.^57^ Previous SCI studies in animal models of SCI have considered TLRs for their role in activating acute intraspinal inflammation, with complex results indicating that studies examining different TLRs in specific cell types at multiple time points post-injury may be needed.^58^ In a mouse thoracic contusion model of SCI, intraspinal mRNA for TLRs 1, 2, 4, 5, and 7 were upregulated, but mice lacking TLR2 or 4 had greater loss of myelin, more astrogliosis, and less functional recovery than wild-type mice.^59^ In a mouse model of SCI, activation of TLR2 through a sterile agonist 1 h post-SCI led to a neuroprotective microglia/macrophage phenotype and genetic ablation of TLR2 led to enhanced axonal damage post-SCI.^60,61^ TLR4 plays a positive role in myelination post-SCI, because it was required for acute oligodendrocyte genesis in naïve mice, whereas mice lacking TLR4 after thoracic contusion SCI had decreased myelin sparing and functional recovery.^62^ Intraspinal injection of the TLR4 ligand, HMGB1, into the low thoracic region of mice resulted in activation of macrophages/microglia and elicited neuroinflammation, whereas exposure to extracellular HMGB1 reduced DRG neurite outgrowth *in vitro*.^63^ In a rat thoracic contusion model of SCI, levels of the protein, HMGB1, an endogenous ligand of TLR4, were elevated in spinal cord explants or tissue acutely and even at 8 weeks post-SCI, where it regulates IL-1 expression.^64^ We recently demonstrated elevated levels of protein in serum of persons with acute or chronic SCI.^19^ HMGB1 has been less well studied at the transcriptional level, but recent studies have shown that its transcription is increased in the spinal cord of mice after thoracic contusion injury.^63^ HMGB1 transcription is also elevated in PBMCs from patients with sepsis and that its transcription is negatively regulated by PPARg.^65,66^ Along with elevated levels of HMGB1 protein, the broad transcriptional activation of the TLR pathway here provides evidence that TLR signaling may be an important mechanism by which chronic inflammation is sustained in persons living with SCI. TLRs are being actively explored as therapeutic targets in cancer, allergy, infectious disease, vaccination, and autoimmune diseases, and there are several TLR directed agents in early phase clinical trials.^53^ In able-bodied populations, exercise downregulates TLR levels on immune cells.^33,67^ Given that breaches in the blood–spinal cord barrier have been shown after infections or stress, whereby neurotoxic inflammatory cells and molecules can access the central nervous system, there is likely to be an impact of elevated systemic inflammation within the injured spinal cord tissue, as well as in the periphery, in persons living with SCI.^31,68^ These data therefore open the possibility of a new direction to explore therapeutic options to control inflammation in persons with SCI.

There are many aspects of this study that require improvement. Age may be a confounding factor in this data set, because although the participants were matched for age at the group level, there were more participants >65 years in the SCI than in the uninjured group (*N* = 7, 1 respectively), and circulating levels of cytokines (e.g., IFN-gamma, IL-6, and IL-10) have been shown to rise with age.^69^ Another limitation of this pilot is that we cannot distinguish between lower gene expression in NK cells and a smaller number or percentage of NK cells in persons with SCI, as has been seen previously.^34–36^ In this study of persons with chronic SCI, limited clinical data were available on events that occurred at the time of initial injury, so it is also possible that changes induced are not attributable to SCI, but to other injuries sustained at that time. Nationally, more than one third of patients with traumatic SCI experience a traumatic brain injury (TBI) at the time of injury.^2^ TBI can also cause systemic immune alterations, including increased susceptibility to pneumonia and systemic inflammation, which may be sustained into the chronic phase.^70–72^ In this study, none of the participants had clinically significant cognitive deficits at the time of participation. However, we did not ask participants directly about other injuries or concomitant TBI at their initial injury. For 20 of 31 participants with SCI for whom office notes were available from the acute or chronic phases, no TBI was noted. Going forward, there is also a need to collect prospective data not only on acute events, but also on body composition and physical activity level, given that metabolic syndrome is common in persons with SCI and may increase inflammatory signaling and decrease NK cell activity, independent of injury level.^27–29,33^ Therefore, the data presented here will be used to design a better and appropriately powered study to replicate and extend these findings by RNA sequencing, to test the injury-level dependence of changes in gene expression, and to examine gene expression changes in specific immune cell subsets. Despite its limitations, this is the largest study of transcriptional changes in persons with chronic SCI, and it provides clear mechanistic links to the immune challenges often observed clinically in persons living with SCI.

## Supplementary Material

Supplemental data
