# Supplementary material for: Persons with Chronic Spinal Cord Injury Have Decreased Natural Killer Cell and Increased Toll-Like Receptor/Inflammatory Gene Expression
Source: J Neurotrauma. 2018 Aug 1;35(15):1819–29. doi: 10.1089/neu.2017.5519 (PMC6033303; doi:10.1089/neu.2017.5519)
Supplement: Supplemental data [file Supp_Table1.zip › Supp_Table1.pdf]

Supplementary Data

Supplementary Table 1.

Each worksheet in the workbook shows genes lists as indicated, including: lists of differentially expressed genes in participants compared by group, for example, by SCI status, level of injury, or neurological injury severity (AIS). A worksheet shows module gene expression data of differentially expressed genes in individuals with SCI at levels rostral to T5 compared to uninjured individuals shown in Figure 2 are included. Cellmix deconvolution data are included, as are the pathway identities from separate bioinformatics platforms.
